# Spatio-temporal evolution and prediction of carbon balance in the Yellow River Basin and zoning for low-carbon economic development

**DOI:** 10.1038/s41598-024-65113-1

**Published:** 2024-06-22

**Authors:** Linlin Dong

**Affiliations:** College of International Hospitality and Tourism Management, Lyceum of the Philippines University-Batangas, 4200 Batangas, Philippines

**Keywords:** Land use carbon balance, PLUS model, Main function area planning, Low-carbon economic development zoning, Yellow River Basin, Ecology, Environmental sciences

## Abstract

Studying the carbon effect of land use in watersheds is important for mitigating global warming, promoting coordinated emission reduction in different regions within the watersheds, and realizing high-quality development of the watersheds. Although a number of scholars have carried out relevant studies in the past, they mainly focused on carbon emissions, rarely involved the carbon balance formed by carbon sources and sinks, and lacked relevant studies on the development of low-carbon economy sub-region. Based on this, this study takes the Yellow River Basin as an example, explores the spatial and temporal evolution of carbon emissions from land use in counties in the Yellow River Basin from 1980 to 2020, and predicts the spatial pattern of carbon income and expenditure from land use under natural conditions in 2030 and 2060 using the PLUS model; and then superimposes on the main functional area planning, divides 735 counties in the Yellow River Basin into six low-carbon economic development subregions, and analyzes their economic development The model of their economic development is analyzed. The results show that: (1) the spatial and temporal differentiation of land use carbon balance in the Yellow River Basin has changed greatly over the past 40 years, (2) the spatial distribution pattern of land use carbon balance in the natural context in 2030 and 2060 is more similar to that in 1990, (3) the carbon emission reduction potentials and pattern optimization of the different low-carbon economic development subregions differ greatly, and they have different low-carbon economic development patterns. The results of this study provide a theoretical basis for scientifically and rationally formulating economic policies for low-carbon development in the counties of the Yellow River Basin, and also provide an important reference for related studies in other similar basins or regions in the world.

## Introduction

Global warming poses a serious threat to ecosystems and human societies, and reducing CO_2_ emissions has become one of the major challenges to human survival and development^1^. Carbon emissions caused by human activities are considered to be the main cause of climate change^2^. While land use change is a concentrated reflection of human activities, urban development has increased the demand for agricultural land and timber products, and the consumption of large amounts of fossil energy, leading to land use transformation, which in turn affects carbon emissions^3^. Therefore, the carbon effect of land use change in the region has become one of the current hot research contents, which has received extensive attention from governments and academics^4^. In response to global warming, China has put forward the strategy of realizing “carbon peak” in 2030 and “carbon neutrality” in 2060. It is of great significance for regional economic and ecological development to make development plans for low carbon development in response to the carbon neutral status.

Since the 1980s, scholars have begun to pay attention to the ecological and environmental benefits caused by land use change, especially the impact of carbon balance of land use change^5^. Many scholars at home and abroad have used land use data in different periods to estimate carbon balance changes^6^. For example, scholars in the United States^7^, Europe^8^ and Brazil^9^ study land use carbon emissions in different periods^10^. The results of related studies also show that land use carbon emissions account for 66% of Brazil's CO_2_ emissions^9^; European countries are adopting measures to change land use patterns and thus increase carbon sequestration, with obvious effects^11^. Rapid urban expansion in China has triggered a series of environmental problems, and thus scholars have assessed carbon emissions directly or indirectly through land use based on elements such as urban agglomeration and population growth^12^. Existing research on land use carbon balance is mainly carried out from both theoretical and empirical aspects. At the theoretical level, scholars have defined the basic connotation and essential characteristics of carbon balance, summarized the basic framework of low-carbon development model^13,14^, and proposed to construct the balance of “carbon source-sink” based on the differences of carbon sources/sinks among regions^15^; at the empirical level, the balance of carbon source-sink has been studied in the areas of forest^16^, wetland^17^, agricultural land^18^, and tourist land^19^. At the empirical level, a lot of research has been carried out on the carbon emission quota allocation mechanism^20^ and regional low-carbon zoning^21^ in the fields of forests^16^, wetlands^17^, agricultural land^18^, and tourism land^19^. Based on this, this paper makes a prediction on the basis of the latest research on land use carbon emissions, and predicts the carbon balance in 2030 and 2060 in the context of the “dual-carbon” target, which is an extension of the current research and effectively plays a role in the future planning of carbon balance at the county scale.

In terms of research scales, previous studies have mainly focused on the national^22^, provincial^23^, city clusters arising from urban agglomeration, and municipal scales^24^. Human activities at different scales cause the expansion of urban land use, which brings a large amount of anthropogenic carbon emissions. This anthropogenic carbon emission is the result of many natural and socioeconomic factors. This provides a study area for the research scope of the county scale in the Yellow River basin, from the county scale, a more accurate understanding of the current county carbon revenue and expenditure situation and low-carbon development policies, compared with the city and provincial scale, the county scale is more microscopic, and for the implementation of low-carbon policy is more efficient. In terms of research methodology, Li et al. discussed the carbon neutralization and spatial autocorrelation through the Moran index, and the geographic detector model was carried out to explore the driving mechanism^6^.Wang et al. analyzed the spatial clustering and influencing factors under the condition of carbon balance generated by land use through the spatial error model, geographically weighted regression model and other regression methods^25^. Scholars comprehensively use the measurement methods such as gray correlation, gravity model, environmental Kuznets curve, and carbon balance concentration index to analyze the relationship between land use carbon balance and other elements, spatial and temporal evolution and driving factors^26,27^. With reference to the latest research results, this paper constructs a land use carbon balance measurement model and applies the measurement method to predict the future development of land use carbon balance, which is a further application and implementation of the measurement analysis of the carbon balance measurement model. The use of the PLUS model to predict the future carbon revenue and expenditure status strengthens the relevance of carbon revenue and expenditure research for future low-carbon development. In terms of territorial spatial zoning, scholars have conducted zoning based on measurement data, policy planning and other methods^28^. In terms of zoning based on metrological data, scholars have carried out systematic zoning through statistical algorithms such as the standard explicit comparative advantage index, K-means clustering, etc., and combined with economic, demographic, and resource factors to zoning low-carbon development^29^. However, it is difficult to match the theoretical planning scope derived only from statistical measurement methods with the actual urban development function orientation^30^. In terms of zoning based on policy planning, the State Council promulgated and implemented the National Main Functional Areas Plan in 2011, which divides the national land space into four types of functional area scopes, namely, key development zones, main agricultural product production zones with restricted development, key ecological functional zones with restricted development, and prohibited development zones, on the basis of the functional differences in regional spatial development^31^. Due to the large difference in development intensity between restricted and non-restricted zones, it leads to a large difference in land use carbon balance between regions. However, previous studies seldom superimposed the main functional areas into the zoning process, which caused a large gap between the zoning and the actual development function zoning. The Yellow River Basin is an energy basin and an important food production base in China^32^. The natural and social environments within the basin vary greatly, and it is one of the regions most affected by human activities^33^. The upper reaches of the basin are an important ecological conservation area, the middle reaches of the basin face problems such as soil erosion and sedimentation, and the lower reaches of the basin suffer from serious ecological damage^34^. Differences in natural ecological environment and socio-economic development level lead to different types of land use, which in turn affects the differences in carbon balance between the upper, middle and lower reaches of the Yellow River Basin. Since the reform and opening up, the rapid expansion of urban land area, the growth of arable land area and the large consumption of energy and fuel brought about by the increased intensity of human activities have led to a large amount of greenhouse gas emissions^35^. Long-term high-intensity resource extraction, the regional resource and environmental carrying capacity is seriously overloaded. However, different socio-economic development conditions in the upper, middle and lower reaches of the Yellow River Basin lead to different urban spaces, and the carbon balance under the influence of social factors is characterized by regional characteristics^36^. Therefore, it is of typical significance to select the Yellow River Basin for the study of the spatial and temporal characteristics of carbon balance, which can help to understand the intensity of different spatial and temporal carbon balance and spatial heterogeneity characteristics, predict the future carbon balance in the Yellow River Basin as well as formulate a scientific and reasonable economic policy for low-carbon development.

Summarizing the literature review, this study takes 735 counties in the Yellow River Basin as the study area to analyze the spatial and temporal evolution of carbon balance and the zoning of low-carbon economic development. The objectives of this study are as follows: (1) to elucidate the spatial and temporal characteristics of carbon emission, carbon absorption and carbon neutrality in the Yellow River Basin at the county scale from 1980 to 2020; (2) to use the PLUS model to predict the status of carbon revenues and expenditures in 2030 and 2060; and (3) to superimpose the planning of the main functional zones under the guidance of the national policy of national territorial spatial planning, and to delineate the low carbon economic zones of carbon revenues and expenditures. In order to provide a theoretical basis for the Yellow River Basin to achieve carbon neutral and low carbon economic development and the optimization of land use structure, as well as to provide a reference for other similar regional watersheds.

## Overview of the study area

**Figure 1:**
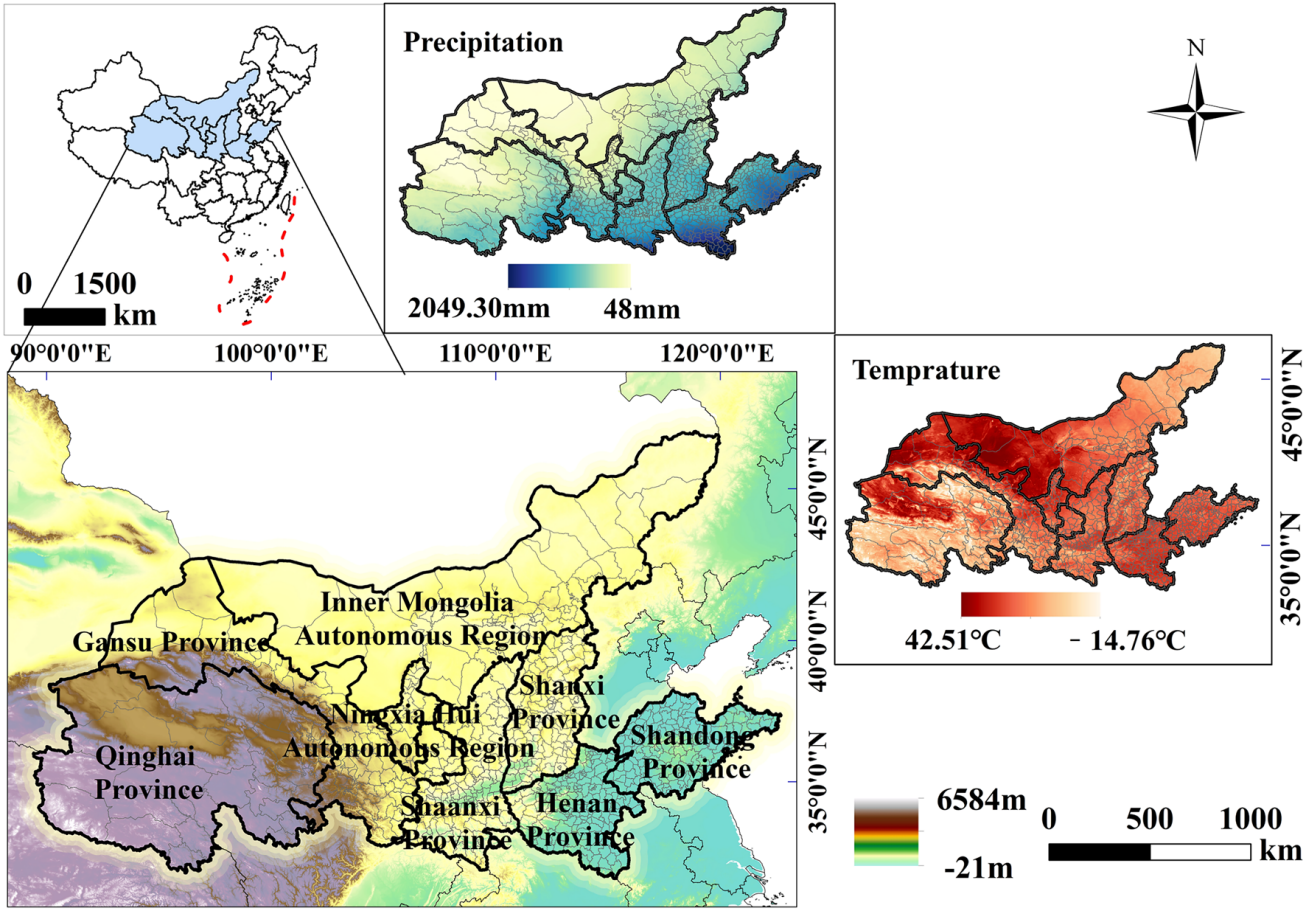
Overview of the YRB study area.

The study area of this paper is the YRB, located between 32°6′53″ N and 41°48′18″ N, 95°50′29″ E–119°06′53″ E. The Yellow River originates from Ba Yan Ka La Mountain in the Qinghai-Tibet Plateau, with a total length of 5464 m. The dividing point between the upper and middle reaches of the Yellow River is Hekou Town, Toketo County, Inner Mongolia Autonomous Region, and the dividing line between the middle and lower reaches is Mengjin County, Luoyang City, Henan Province^36^. The YRB covers an area of about 3.6 × 10^6^ km^2^, spanning three terrain steps and the eastern, central and western economic zones, and is an important ecological barrier and economic zone in China (Fig. 1). The Wei River and Fen River are first-class tributaries of the YRB, and the basin shows obvious geospatial heterogeneity characteristics. The YRB is located in the mid-latitude zone, with plateau mountain climate and temperate continental climate dominating in the upper reaches, and temperate monsoon climate dominating in the middle and lower reaches^37^. The temperature difference within the basin is obvious, with the average annual temperature ranging from −4 to 14 °C, the longitudinal zonation is significant, the average annual precipitation is 200–600 mm, and there is a big difference in precipitation within the basin, which gradually decreases from the southeast to the northwest. The variability of climate, topography and soil in the YRB has created a rich variety of vegetation types.

The upper reaches of the Yellow River are sparsely populated, while the middle and lower reaches are densely populated with high intensity of human activities. In 2022, the urbanization rate of the YRB (61.30%) is lower than that of the whole country (65.20%) by about 3.9 percentage points, and there are large regional differences in urban development. Among the various land use types, grassland has the largest share of area (40.59%), followed by unutilized land (27.30%), cultivated land (18.12%), and inpermeable surface (3.23%). Reference to relevant research^38^. In this study, 735 county-level administrative units in the basin were selected as the scope of the YRB.

## Data sources and methods

### Research framework

**Figure 2:**
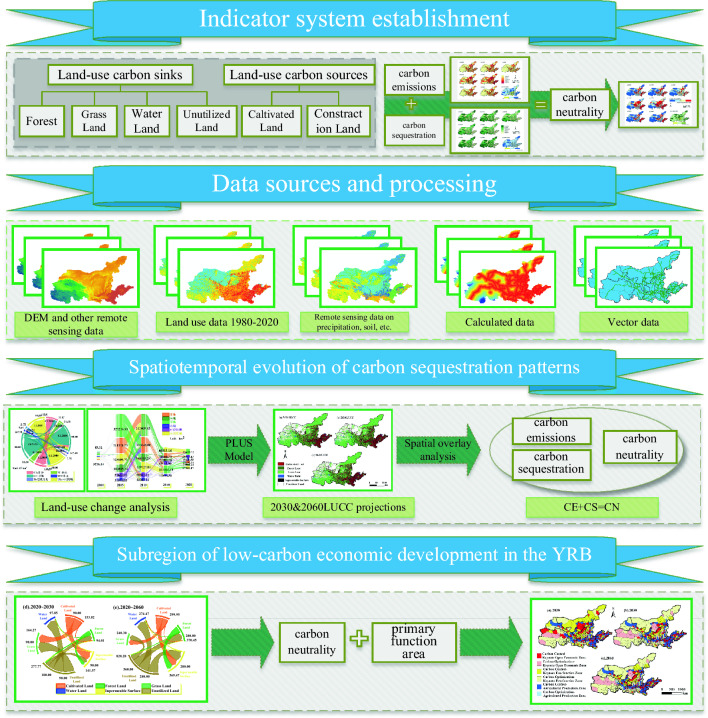
Research framework diagram.

This paper aims to study the spatial and temporal evolution of carbon balance in the Yellow River Basin and the zoning of low-carbon economic development with the goal of “two-carbon”, and the research framework of this paper is divided into four parts (Fig. 2). The first part is to construct a land-use carbon balance model at the county scale in the Yellow River Basin, with arable land and construction land categorized as carbon sources, and forests, grasslands, water bodies and unused land categorized as carbon sinks. Overlaying the administrative area vector data for mask extraction, the land use data of the study area with 735 administrative units as boundaries are obtained. The second part analyzes the processing and steps of various data and research methods. The third part is to analyze the spatial and temporal evolution of carbon emission, carbon sequestration, and carbon neutrality in the Yellow River Basin from 1980 to 2020, and on the basis of which, the land use status of the Yellow River Basin in 2030 and 2060 is simulated and predicted. The fourth part is to carry out the zoning of low carbon economic development in the Yellow River Basin. According to the results of carbon emission, the carbon neutral space of the Yellow River Basin is divided into carbon control zones and carbon optimization zones, and the main functional zones select the key development zones, the main production zones of agricultural products that are restricted from development, and the key ecological functional zones that are restricted from development as the main basis for categorization. According to the carbon neutral zoning superimposed on the main functional area, a total of six low carbon economic zoning categories of counties in the Yellow River Basin are classified.

### Data sources and processing

The 1980, 1990, 2000, 2010 and 2020 land use data of the YRB used in this study were obtained from the Center for Geo-Resource Science, Environment and Data, Chinese Academy of Sciences (CAS) (https://www.resdc.cn/). The data set is based on remotely sensed monitoring data with a spatial resolution of 30 m. This paper reclassifies five periods of remote sensing monitoring data using county-level administrative boundaries of the YRB, and reclassifies the land use data into six categories of land use types, such as cropland, forest land, grassland, watersheds, The 1980, 1990, 2000, 2010 and 2020 land use data of the YRB used in this study were obtained from the Center for Geo-Resource Science, Environment and Data, Chinese Academy of Sciences (CAS) (https://www.resdc.cn/). The data set is based on remotely sensed monitoring data with a spatial resolution of 30m. This paper reclassifies five periods of remote sensing monitoring data using county-level administrative boundaries of the YRB, and reclassifies the land use data into six categories of land use types, such as cultivated land, forest land, grassland, water build, inpermeable surface and unutilized land. Then, this study superimposed the administrative district vector data for mask extraction to obtain land use data bounded by 735 administrative units in the study area. The 2030 and 2060 land use data used in this study were obtained from projections using the PLUS model and unutilized land. Then, this study superimposed the administrative district vector data for mask extraction to obtain land use data bounded by 735 administrative units in the study area. The 2030 and 2060 land use data used in this study were obtained from projections using the PLUS model.

County administrative vector boundaries are derived from the standard map service system (http://bzdt.ch.mnr.gov.cn/). In ArcGIS 10.8, the counties were selected according to the name of the administrative regions, and then the counties within the boundaries with the administrative regions were selected for extraction, and finally 735 county administrative vector maps were obtained. The socio-economic data were obtained from the Statistical Yearbook of Prefectural Cities, City Yearbook and County Statistical Yearbook of National Bureau of Statistics of the YRB (http://www.stats.gov.cn/), etc. The data on the zoning of main functional areas are derived from the Provincial Planning of Main Functional Areas (https://www.gov.cn/). Corresponding correlation of county functional zoning text and county main functional attributes in the main functional area planning, using ArcGIS10.8 software, changing the attribute symbols statistically presenting the spatial visualization of the main functional area. Reference to previously published articles and repeated validation^39,40^, Data of different pixel sizes were processed through ArcGIS 10.8 software, and all spatial data were unified in the projected coordinates: WGS_1984_World_Mercator. In this study, different land use data are superimposed on administrative zoning and main functional area zoning, and a zoning statistics tool is used to calculate the number of rasters for land use categories within the administrative area.

### Methodology of the study

#### Carbon balance measurement modeling

Carbon sources and sinks are the main forms of land use carbon emission measurement^41^. Referring to related studies^27^, cultivated land and inpermeable surface are carbon sources, and forest land, grassland, water build and unutilized land are carbon sinks^18–22^, which are calculated by the following formulas:

1 $$E = \sum {e_{i} = \sum {S_{i} \times V_{i} } }$$

In Eq. (1), *E* is the total amount of carbon emissions; *Si* is the area of a certain land use type; and *V*_*i*_ is the carbon emission coefficient of a certain land type. The carbon emission coefficients of cultivated land, forest land, grassland, water build and unutilized land are 0.4970, −0.5810, −0.0205, −0.0253 and −0.0005 t hm^−2^, respectively. Carbon emissions from built-up land mainly include urban green space, fossil, electricity consumption, etc^42^.

2 $$E_{c} = \sum {E_{i} \times f_{i} }$$

In Eq. (2), *Ec* is the total carbon emissions from construction land; *E*_*i*_ is the consumption of each energy source, *f*_*i*_ the emission coefficient of each energy source. Based on the IPCC Carbon Calculation Guidelines and related research results^43,44^, The carbon emission factors for coal, oil, and natural gas are 0.748 t t^−1^ standard coal, 0.583 t t^−1^ standard coal, and 0.444 t t^−1^ standard coal, respectively.

Carbon emissions in the region are equal to the sum of the total carbon emissions from land-use type transformation and the carbon emissions from energy consumption on inpermeable surface, *A *= *E *+ *Ec*, with A being the total carbon emissions in the region; *E* the total carbon emissions from land-use types; and *Ec* the total carbon emissions from inpermeable surface.

#### PLUS model

The PLUS model better simulates changes in multiple land use categories at the patch level compared to spatial prediction models such as artificial neural networks, FLUS, and CLUE-S models^45^. Therefore, in this study, the PLUS model was chosen to simulate future land use scenarios (2030 and 2060) under the condition of adding multiple land use drivers. The specific steps are as follows:

First, the land use data is converted to uc format. Twelve land use drivers such as DEM, slope, soil type and precipitation as natural factors, population, GDP, impervious cover and transportation accessibility elements (distance to highways, primary and secondary roads, high speed rail lines, etc.) as socio-economic factors, and watershed conditions as constraints were selected and analyzed using the PLUS model LEAS module^27^.

Then, the predicted 2020 land use was tested for accuracy against the actual land use, and the correlation coefficient settings were summarized (Table 1), with a Kappa coefficient of 0.9245. The test results showed that the predictions were credible and the model had the ability to accurately simulate and predict future land use.

**Table 1:** The number of land use rasters predicted by the PLUS model for 2030 and 2060.

	Cultivated land	Forested land	Grassland	Watershed	Construction land	Unutilized land
2020	7,906,971	3,586,264	18,670,703	1,076,979	1,415,024	12,869,672
2030	7,736,057	3,600,947	18,853,227	1,185,697	1,554,546	12,595,139
2060	7,573,690	3,639,252	18,937,700	1,381,950	1,998,261	11,994,760
Related parameter settings	Neighbourhood size: 3					
	Thread: 5					
	Patch generation threshold: 0.5					
	Expension coefficient: 0.1					
	Percentage of seeds: 0.001					
Kappa coefficient	0.9245					

Finally, based on the results of the development probability calculations, the watershed area will be used as a limiting conversion factor to predict the land use types using the PLUS model. In this study, the Marcov Chain will be used to predict the total amount of each type of land use in 2030 and 2060, respectively, and imported into the multi-class stochastic patch seed module of the PLUS model for CARS simulation of the land use results under the traditional scenarios in 2030 and 2060.

#### Low-carbon economy zoning methodology

Based on the theoretical concept and framework of territoriality^46^, Construct a spatial zoning of low-carbon economy under the perspective of main functional areas, comparing the carbon balance superimposed on the main functional areas in 2020, 2030 and 2060. Divide the study area counties into two zones, carbon control zone and carbon optimization zone, based on carbon neutrality^47^.

The main functional zones under the territorial spatial planning will be strategically oriented functional zoning of each province, which is mainly divided into four categories: key development zones at the national and provincial levels, main agricultural production zones with restrictions on development, key ecological functional zones with restrictions on development, and prohibited development zones^24,32^. Among them, the key development zones are urbanized areas that provide mainly industrial and service products at the provincial or national level; and the main agricultural production zones that are restricted from development are areas that provide food security and supply of agricultural products at the provincial or national level; The key ecological function areas that restrict development are the functional areas that provide the main body of ecological services at the provincial level that have been recognized at the national level; The basic unit of the natural or legal boundaries of the prohibited development zones, distributed in other main functional zoning counties (districts) at all levels of nature reserves, cultural heritage, forests and geoparks, scenic spots, etc., according to the relevant laws need to implement mandatory protection of the prohibited development zones. Therefore, this study does not consider the status of low-carbon economic zoning in the prohibited development zones^48^.

The main functional area selects the key development zones, the main production areas of agricultural products that are restricted from development and the key ecological function zones that are restricted from development as the main classification basis^49^. Based on the carbon neutral zoning superimposed on the main functional zones, the low carbon economic zoning is divided into: carbon control—key development zones (CC-KDZ), carbon control—agricultural products main production zones (CC-APZ), carbon control—key ecological functional zones (CC-KEZ), carbon optimization—key development zones (CO-KEZ), carbon optimization—agricultural products main production zones (CO-APZ), and carbon optimization—key ecological functional zones (CO-KEZ), totaling six levels of low carbon economic zoning for counties in YRB.

## Results

### Characteristics of land-use change in the YRB

#### Characteristics of land use change, 1980–2020

**Figure 3:**
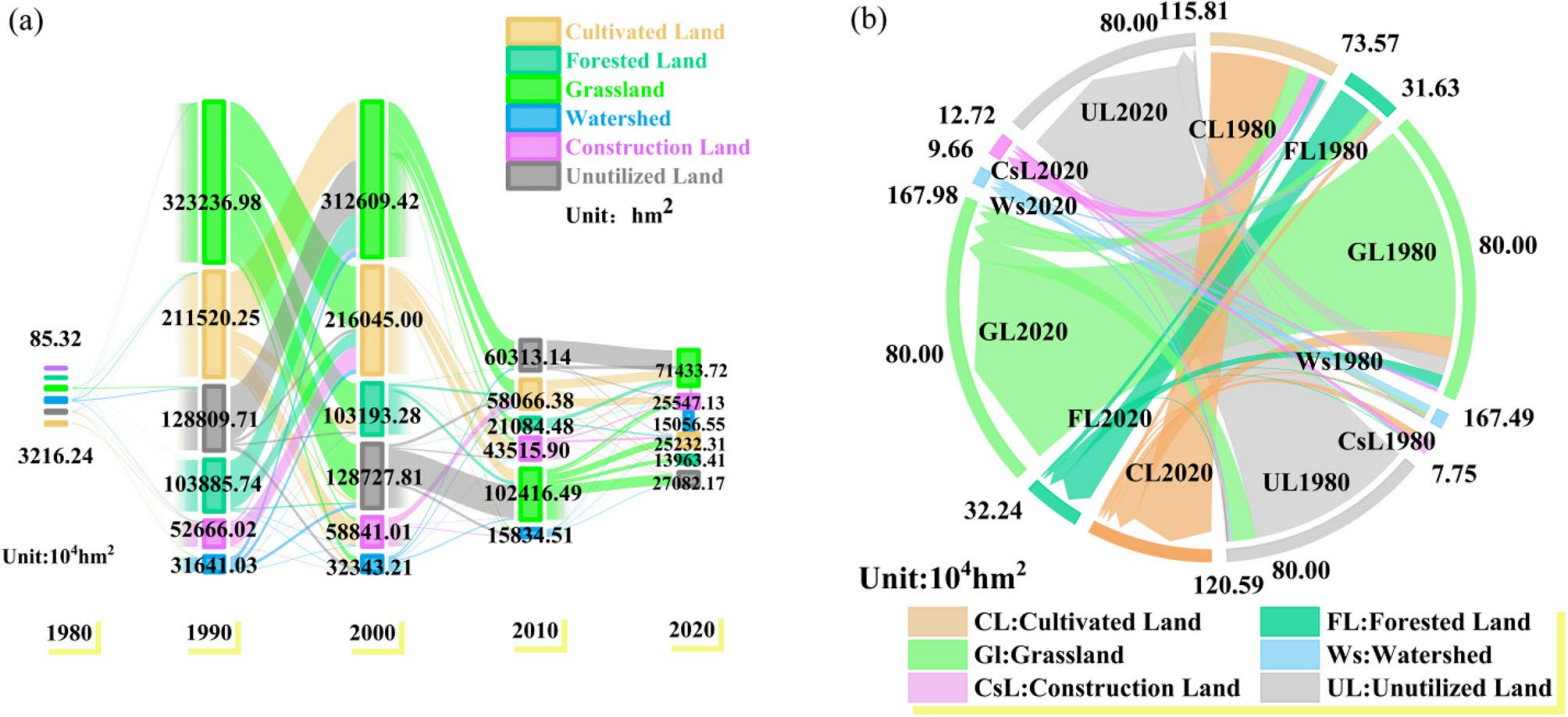
Land use changes in the YRB from 1980 to 2020. **(a**) Matrix of land use transfer in the YRB in 1980, 1990, 2000, 2010 and 2020, (**b**) total area of land use transfer in the YRB in 40 years for various types of land uses.

In this paper, the land use of the five periods of 1980, 1990, 2000, 2010 and 2020 are superimposed and analyzed to obtain the distribution pattern of land use transfer (Fig. 3a) and the overall transfer area of each land use category (Fig. 3b).

During the period of 1980–1990, the number of transfer of various types of land use changes was small, and the direction of land outflow was concentrated in the transfer of cultivated land to inpermeable surface (1893.06 hm^2^), the transfer of water bodies to cultivated land (1894.59 hm^2^) and inpermeable surface (1247.31 hm^2^), and the net transfer of various types of land to inpermeable surface reached a maximum area of 3788.01 hm^2^. In 1990–2000 and 2000–2010, the change of land-use transfer became larger, and the transfer area of all kinds of land to inpermeable surface reached 3.18 × 10^4^ hm^2^ during the period. 1980–2000, waters were transferred to cultivated land and inpermeable surface, and forest land was transferred to unutilized land, and the reverse transfer began to appear in 2000–2010, with all kinds of land transferred to forest land (1.59 × 10^4^ hm^2^ ) and waters (5152.14 hm^2^ ), while the net transfer area of all kinds of land to inpermeable surface reached 2.56 × 10^4^ hm^2^ , of which cultivated land was transferred to inpermeable surface. In 2000–2010, a reverse transfer began to occur, with various types of land being transferred to forest land (1.59 × 10^4^ hm^2^) and water (5,152.14 hm^2^), while the net transfer of various types of land to inpermeable surface amounted to 2.56 × 10^4^ hm^2^, with the net transfer of cultivated land to inpermeable surface amounting to 1.76 × 10^4^ hm^2^. From 1990 to 2010, the net transfer area from grassland to unutilized land was 15.44 × 10^4^ hm^2^, which was much larger than the transfer area between other types of land. From 2010 to 2020, the land use changes were mainly concentrated in the transfer of all types of land to inpermeable surface, with a net transfer area of 1.77 × 10^4^ hm^2^, and the transfer of arable land to grassland and unutilized land before 2010 was reversed, with a net transfer area of 1.03 × 10^4^ hm^2^ from all types of land to waters in the period of 2010–2020. In 2020, there is a reverse transfer, with a net transfer of cultivated land to grassland of 4009.86 hm^2^ and a net transfer of all types of land to water of 1.03 × 10^4^ hm^2^.

Changes in the total amount of land-use transfer from 1980 to 2020 show a net decrease in the area of cultivated land of 2.45 × 10^4^ hm^2^, a net increase in forest land of 6086.07 hm^2^, a net increase in grassland of 4885.56 hm^2^, a net increase in water build of 1.15 × 10^4^ hm^2^, a net increase in the area of land used for construction of 4.98 × 10^4^ hm^2^, and a net decrease in the area of unutilized land of 4.78 × 10^4^ hm^2^. The biggest change in the amount of net transfer of land use from inpermeable surface and unutilized land was recorded, with the amount of transfer of all types of land to inpermeable surface reaching 12.42 × 10^4^ hm^2^ and the area of inpermeable surface expanding. The net transfer of cultivated land and unutilized land to other types of land use reached 8.10 × 10^4^ hm^2^ and 4.78 × 10^4^ hm^2^, a larger amount. Grassland has the largest amount of area transferred into and out of the country, with an average transfer area of 1.45 × 10^4^ hm^2^, while the amount of net transferred area is much smaller than the area transferred into and out of the country.

#### Spatial pattern of land use change in 2030 and 2060

**Figure 4:**
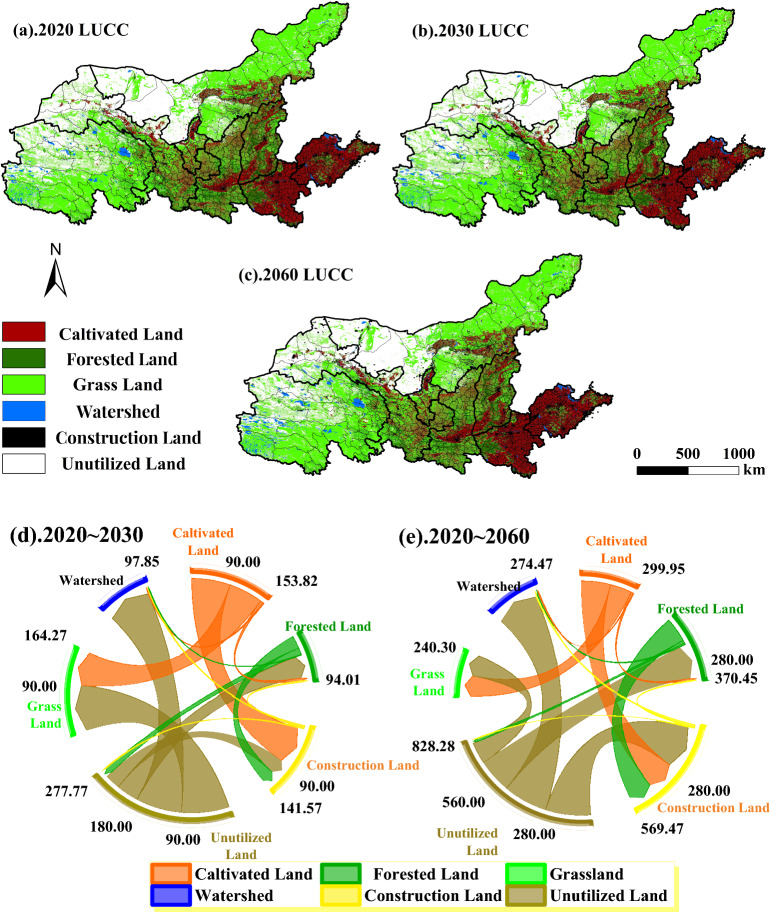
Projections of land use types under natural scenarios in 2030 and 2060. (**a**) Distribution of land use scenarios in 2020, (**b**) projected land use scenarios under natural scenarios in 2030, (**c**) projected land use scenarios under natural scenarios in 2060, (**d**) transfer of land leases in 2020–2030, (e) land use transfers in 2020–2060 land use transfer scenario.

In this study, the spatial pattern of land use in the YRB in 2030 and 2060 is predicted using the CA module of the PLUS model (Fig. 4), and the total area and percentage of each type of land use are analyzed.

In this study, the PLUS model was used to predict the land use in 2030 and 2060 under the natural scenario (Figs. 3b, 4c). The status of the share of each type of land use is the same as in 2020 (Fig. 4a), with grassland having the largest share of the area.The area of grassland in 2030 and 2060 is 16,967.9 × 10^4^ hm^2^ and 17,043.93 × 10^4^ hm^2^ , with a share of 41.41% and 41.60%, respectively. It is followed by unutilized land, which accounts for 27.67% and 26.36% respectively, and the rest of the land use area is in the order of cultivated land > forestland > inpermeable surface > water build. The land use change in 2020–2030 (Fig. 4d) shows that the net transfer out of cultivated land is 153.82 × 10^4^ hm^2^, of which, the area shifted to grassland and inpermeable surface is more, 68.43 × 10^4^ hm^2^ and 77.33 × 10^4^ hm^2^, respectively; the area shifted out of forest land is 40.40 × 10^4^ hm^2^, and the net decrease of forest land is 34.90 × 10^4^ hm^2^. The area of inpermeable surface increased the most to 133.57 × 10^4^ hm^2^, of which, the area of cultivated land converted to inpermeable surface accounted for 57.89%. The largest area of unutilized land was transferred out, amounting to 262.43 × 10^4^ hm^2^, of which more was shifted to grassland and water, accounting for 71.28%.

In 2020–2060, the area of unutilized land transferred out reaches 807.85 × 10^4^ hm^2^, and the net area transferred out reaches 787.42 × 10^4^ hm^2^, which is the largest amount, and all types of land, except cultivated land, have unutilized land transferred into them (Fig. 4e). The net transfer out of arable land was 299.95 × 10^4^ hm^2^, of which 94.31% was transferred to grassland and inpermeable surface. The net transfer of inpermeable surface is 524.91 × 10^4^ hm^2^ , which is second only to the amount of unutilized land transfer. In conclusion, relative to the land use status in 2020, the number of land use transfers in 2060 are all greater than the number of transfers in 2030, with the highest number of net transfers out of unutilized land, the highest number of net transfers in from inpermeable surface, and the smallest changes in watersheds and grasslands.

### Analysis of carbon balance in the YRB

In this study, the spatial and temporal distribution of carbon emissions, carbon sequestration and carbon neutrality in the YRB from 1980 to 2020, 2030 and 2060 were automatically classified based on the natural discontinuity method in ArcGIS. Among them, carbon emission and carbon absorption are categorized into five levels, including Lowest, Lower, Moderate, Higher and Highest. Carbon neutral spatial zoning is divided into: Carbon Control Zone (Carbon Emission > Carbon Absorption) and Carbon Optimization Zone (Carbon Absorption > Carbon Emission).

#### Spatial and temporal evolution of carbon emissions

**Figure 5:**
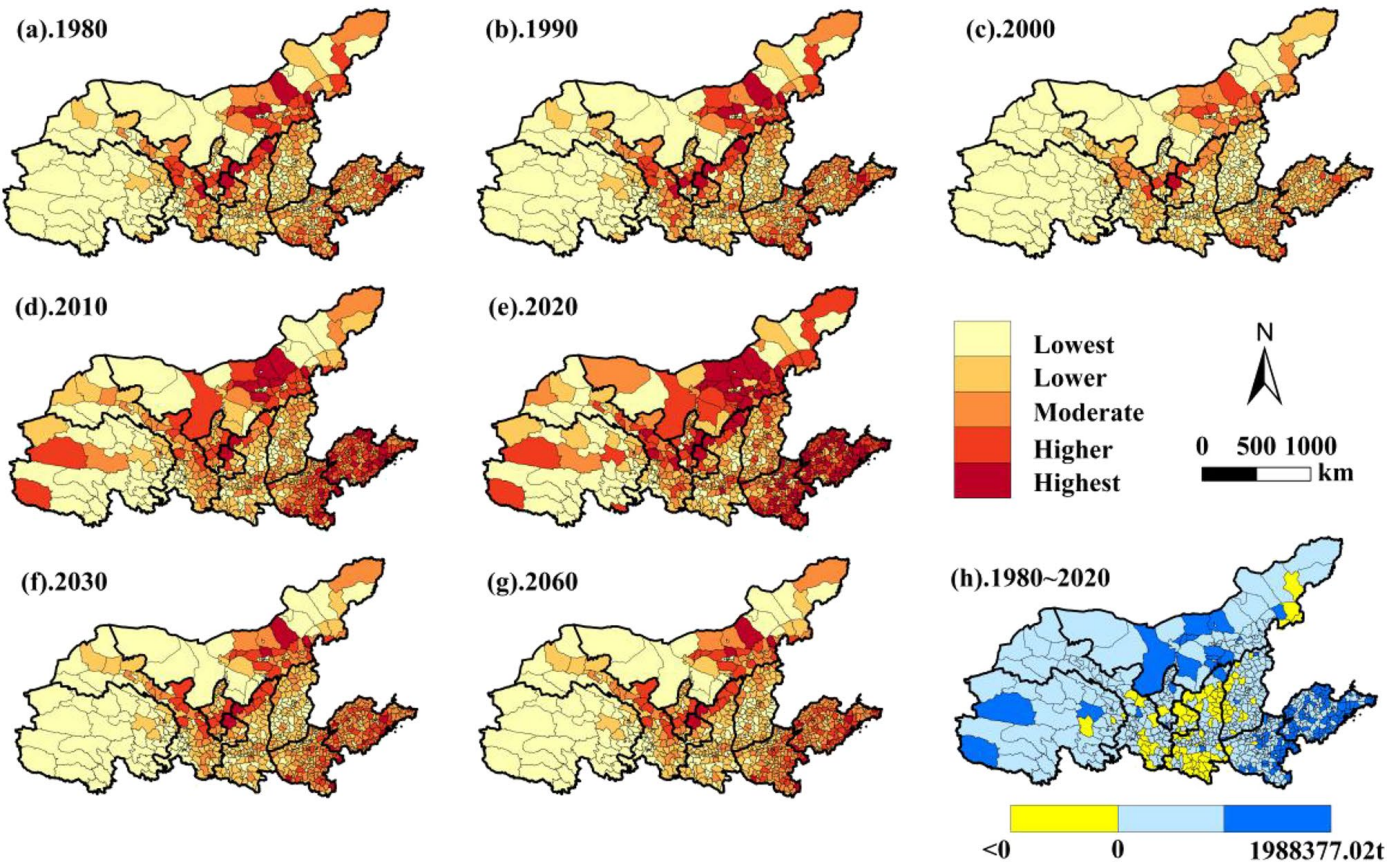
Spatial and temporal evolution of carbon emissions in the YRB.

From 1980 to 2020, the total carbon emissions in the YRB changed considerably (Fig. 5). In time, the total carbon emission decreased from 4.10 × 10^8^ t in 1980 to 3.15 × 10^8^ t in 2000, and then increased to 7.31 × 10^8^ t (2020), an increase of 56.09% compared with 1980. Meanwhile, the average value of carbon emission in each county (district) area decreased from 55.80 × 10^4^ t (1980) to 42.91 × 10^4^ t (2000), and then increased to 99.45 × 10^4^ t (2020), an increase of 56.11%. This shows that the total carbon emissions and carbon emissions of counties (districts) in the whole YRB have changed greatly, and both show a "V" type growth trend.

During the past 40 years, carbon emissions in the YRB increased in a total of 637 counties, accounting for 86.79% of the number of counties in the YRB, with the highest being Lanshan District and Huangdao District, which increased by 198.84 × 10^4^ t and 196.27 × 10^4^ t respectively, and were discretely located in the municipal districts of Shandong Province, the municipal districts of Henan Province, and counties of the Dalat Banner and the Jungar Banner in Inner Mongolia. Carbon emissions decreased in only 98 counties, concentrated in the north and south of Shaanxi Province, the south-east of Gansu Province, and discretely distributed in the east of Inner Mongolia, the Luliang area of Shanxi Province and around Qinghai Lake. Spatially, the carbon emissions of the counties in the YRB show obvious east–west and north–south differences. And with the increase of time, the carbon emissions of each region gradually increase, and the high value area is gradually dense. From discrete distribution in 1980 in 10 counties such as Huining County, Huanxian County, Ulatqian Banner and Shenmu City, there is a concentration in 2020, and the centralized centers are distributed in Shandong, Henan, the central counties and cities of Inner Mongolia, and some counties and cities of the Hexi Corridor, and decreasing to the surroundings. Topographically, high carbon emission areas are mainly concentrated in the hilly areas of the Shandong Peninsula, the central and eastern plains of Henan Province, the urban centers of central Inner Mongolia, the Hetao Plain, and the plateau areas of the Hexi Corridor.

The projections of carbon emissions from land use in 2030 and 2060 under the natural scenario found that the total carbon emissions in 2030 and 2060 amounted to 4.51 × 10^8^ t and 4.63 × 10^8^ t, respectively; compared with 2020, there was a reduction of 62.08%, 57.89%, respectively. Changes in carbon emissions have stabilized and are less variable. The average values of carbon emissions from counties in the YRB in 2030 and 2060 are 61.33 × 10^4^ t and 62.92 × 10^4^ t, respectively, indicating that the carbon emissions from counties in the YRB change less with time. In terms of spatial distribution, the spatial distribution of land-use carbon emissions in 2030 and 2060 is similar to that of 2000, the early stage of industrialization development. High-value areas of carbon emissions are discretely distributed in central Shandong Province, central and eastern municipal districts of Henan Province, the Hexi Corridor region, and cities in central Inner Mongolia. Agricultural farming areas and urban development areas along the main stream of the Yellow River have the highest carbon emissions, while the Three Rivers source area of the Qinghai-Tibet Plateau, the grassland areas in eastern and western Inner Mongolia, and the Taihang Mountains and the Qinling-Huaihe River areas have lower carbon emissions.

In terms of the levels of carbon emissions of counties in the YRB (Table 2), from 1980 to 2000, the number of counties at the lowest level was the highest, followed by the Lower level, with the proportion of counties reaching 33.06%. Starting in 2010, the number of counties at the Lower level exceeded the number of counties at the lowest level. It is noteworthy that in 2020, the number of counties at all levels is relatively even, and the number of counties at highest level is greatly increased compared with the previous years. This indicates that the carbon emissions in the YRB, not only the total emissions show an increasing trend, but also the carbon emissions of the county range covered by each level show an increasing trend. Through the prediction of the number of counties at each level of carbon emissions in 2030 and 2060, it is found that the number of counties at Lowest and Lower levels is the largest (477 counties), and the number of counties with carbon emissions at Highest level decreases (only 14 counties) and accounts for the lowest percentage (1.90%). This indicates that the future development trend of carbon emissions in counties in the YRB is that the total amount of carbon emissions decreases while the level of carbon emissions in each county decreases.

**Table 2:** Number and share of counties in various stages of carbon emissions in the YRB.

Level	Number and percentage of counties (%)													
	1980	Proportion	1990	Proportion	2000	Proportion	2010	Proportion	2020	Proportion	2030 projection	Proportion	2030 projection	Proportion
Lowest	262	36.65	251	34.15	355	48.30	186	25.31	130	17.69	221	30.07	211	28.71
Lower	243	33.06	228	31.02	258	35.10	231	31.43	184	25.03	256	34.83	255	34.69
Moderate	168	22.86	181	24.63	104	14.15	158	21.50	147	20.01	170	23.13	171	23.27
Higher	51	6.93	59	8.03	17	2.31	115	15.64	151	20.54	74	10.07	81	11.02
Highest	11	1.50	16	2.17	1	0.14	45	6.12	123	16.73	14	1.90	17	2.31
All	735													

#### Temporal and spatial evolution of carbon sequestration patterns

**Figure 6:**
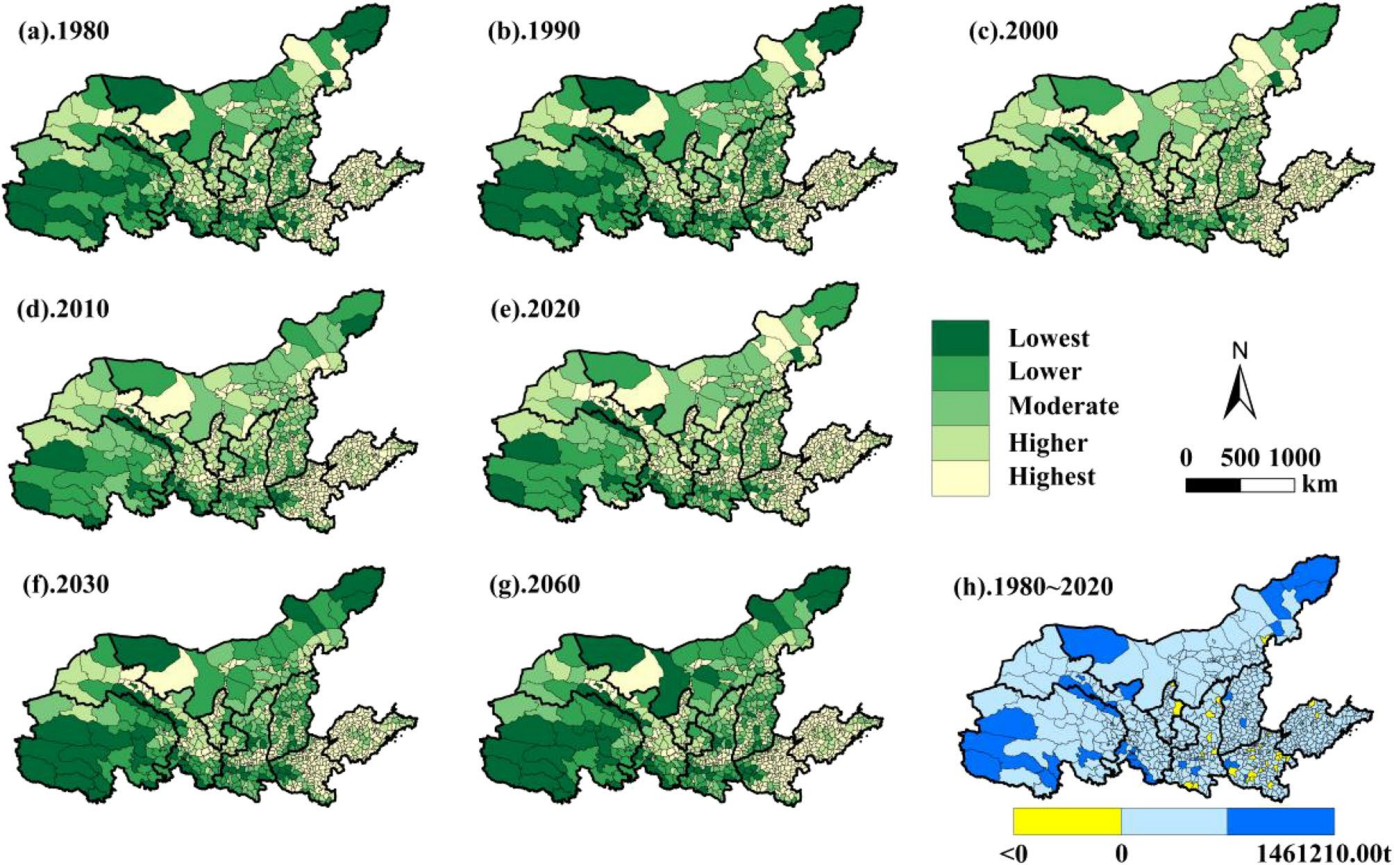
Spatial and temporal evolution of carbon sequestration in the YRB.

The total carbon uptake in the YRB changed relatively little from 1980 to 2020 (Fig. 6). In time, it decreased by 33.78% from 1980 to 2000 (1.44 × 10^8^ t), and showed a steady decreasing trend from 2000 to 2020. The average value of carbon uptake in the counties (districts) of the YRB decreased from 30.24 × 10^4^ t in 1980 to 19.65 × 10^4^ t in 2000, and then changed less until 2020 (20.05 × 10^4^ t). This indicates that from 1980 to 2020, the carbon uptake of counties in the YRB has changed less relative to carbon emissions and is more stable.

During the past 40 years, the counties in the YRB where the total carbon sequestration increased by a total of 40.73 × 10^4^ t were mostly concentrated in the urban fringe, including 44 counties such as Maiyang County, Minquan County, Luochuan County and Wubao County. The remaining 691 counties had a total decrease in carbon sequestration of 0.75 × 10^8^ t, which was much larger than the increase in carbon sequestration. Spatially, carbon uptake in counties showed east–west and north–south differences opposite to carbon emissions. The high value areas of carbon uptake are concentrated in the central and western parts of Qinghai Province, the eastern and western parts of Inner Mongolia, the southeastern part of Gansu, and the southern part of Shaanxi. In terms of topography, they are found in the Sanjiangyuan area of the Qinghai-Tibet Plateau, the Qilian Mountain area at the border of Qinghai and Gansu, the Qinling Mountain area in southern Shaanxi Province, the Taihang Mountain area at the border of Shanxi and Henan, and the grassland area in eastern and western Inner Mongolia. The mountainous terrain area with high vegetation cover is an important carbon absorption zone in the YRB. The high carbon absorption areas and the high carbon emission areas show opposite distribution characteristics in terms of space and topography.

In terms of the various levels of carbon sequestration in the counties of the YRB (Table 3), from 1980 to 2000, the number of counties at the lowest level was the highest, accounting for 54.56%. It is followed by the lower level, with the number of counties accounting for 33.06%, followed by the intermediate (14.01%), higher (12.97%) and the highest (5.17%), and the carbon uptake of the county range covered by each level gradually decreases with small changes. The prediction of the number of counties at each level of carbon sequestration in 2030 and 2060 reveals that the percentage of the number of counties covering each level gradually decreases in 2030 and 2060. This indicates that the future development trend of carbon absorption in counties in the YRB is that the carbon absorption levels in each county are relatively stable over time.

**Table 3:** Number and proportion of counties in each stage of carbon sequestration in the YRB.

Level	Number and percentage of counties (%)													
	1980	Proportion	1990	Proportion	2000	Proportion	2010	Proportion	2020	Proportion	2030 projection	Proportion	2030 projection	Proportion
Lowest	341	46.39	342	46.53	401	54.56	398	54.15	399	54.29	348	47.35	359	48.84
Lower	162	22.04	157	21.36	166	22.58	163	22.17	161	21.90	144	19.59	132	17.96
Moderate	99	13.47	103	14.01	96	13.06	101	13.74	100	13.61	109	14.83	113	15.37
Higher	95	12.93	94	12.79	53	7.21	54	7.35	56	7.61	94	12.79	87	11.84
Highest	38	5.17	39	5.31	19	2.59	19	2.59	19	2.59	40	5.44	44	5.99
ALL	735													

#### Spatial and temporal evolution of carbon neutrality

**Figure 7:**
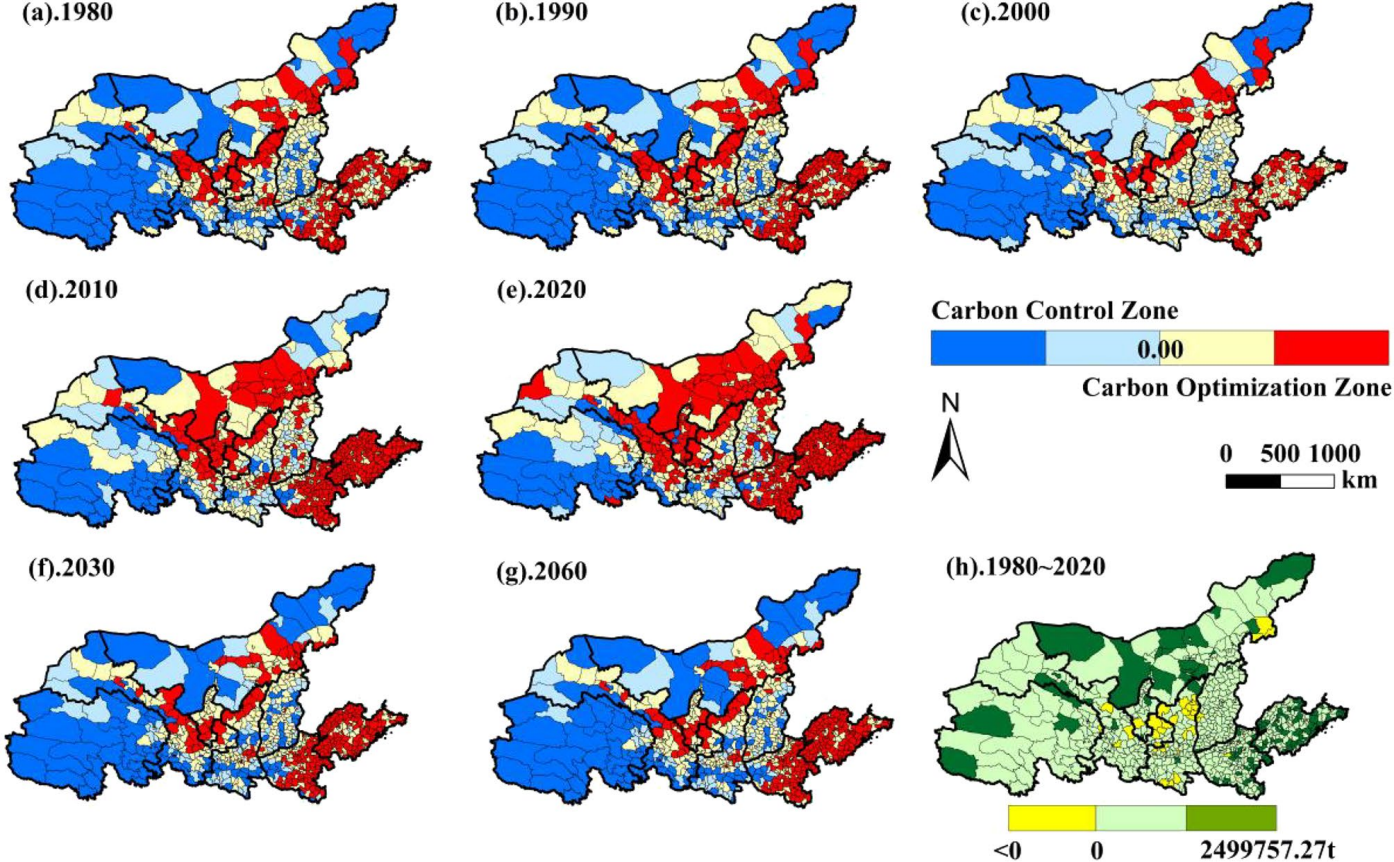
Spatial and temporal evolution of carbon neutral zoning in the YRB.

In this study, the carbon measurement model was used to measure the carbon neutrality status from 1980 to 2020 and predict the carbon neutrality of land use in 2030 and 2060 under the natural scenario, and the carbon neutrality changes were divided into carbon control and carbon optimization zones (Fig. 7).

The total net carbon emissions from 1980 to 2020 change considerably, and the total net carbon emissions in the carbon control zone increase from 1.88 × 10^8^ t in 1980 to 6.24 × 10^8^ t in 2020, an increase of 3.32 times. The total net carbon absorption in the carbon optimization zone decreases from 0.97 × 10^8^ t in 1980 to 0.41 × 10^8^ t in 2020, a decrease of 42.27%, and the net carbon absorption is much smaller than the net carbon emission. In the past 40 years, only 36 counties, accounting for 4.90% of the total number of counties, saw an increase in net carbon uptake, mainly including Taimusi Banner and Zhenglan Banner in the eastern part of Inner Mongolia, some counties in the central and northern edges of Shaanxi Province, the northeastern edge counties of Henan Province and the northern edge counties of Shandong Province. There are 699 counties with an increase in total net carbon emissions, with the largest net carbon emissions reaching 249.98 × 10^4^ t, and the high value areas are distributed in the urban fringes of Huanxian County, Duolun County, Zicang County, and Luochuan County.

Spatially, the area of carbon control areas is much larger than that of carbon optimization areas, and shows obvious spatial heterogeneity characteristics. Carbon control areas are mainly distributed in Shandong and Henan provinces, central Inner Mongolia, Ningxia and central Gansu. Carbon optimization areas are mainly distributed in Qinghai Province, eastern and western Inner Mongolia, Lvliang City in western Shanxi, and central and southern Shaanxi. In terms of topography, the carbon control area is mainly distributed in the hilly area of Shandong, the plain area in the east of Henan, the valley area distributed along the Fen River in Shanxi, the central part of the Inner Mongolia Plateau and the Hetao Plain area, and the Hexi Corridor area in Gansu. Carbon optimization areas are mainly concentrated in the Qinghai-Tibet Plateau area, the Taihang Mountains and the Feniu Mountains, and the Weihe River valley floor.

In terms of the change in the number of carbon control areas and carbon optimization areas in the counties of the YRB, the carbon optimization areas decreased from 158 counties in 1980 to 90 counties in 2020, with the number of counties covered decreasing by 9.25%. The number of counties in carbon control areas increases and is much more than the number of carbon optimization areas. The results of carbon sequestration projections for 2030 and 2060 show that the carbon control areas in the central part of Inner Mongolia and the central part of Shanxi are transformed into carbon optimization areas, and the spatial distribution of carbon control areas is basically the same as that of the carbon control areas in 1990. This indicates that the future spatial development trend of carbon control areas and carbon optimization areas in the YRB counties will be: the carbon optimization areas will gradually increase, the net carbon uptake will increase, and the goal of carbon neutrality will be gradually achieved.

### Sub-region of low carbon economic development in the YRB

#### Results of the low-carbon economic development subregion

This study compares the low-carbon zoning of carbon control zones and carbon optimization zones in 2020, 2030 and 2060, superimposed on the planning of main functional zones, and divides the low-carbon economic zoning under the perspective of main functional zones into six types (Table 4).

**Table 4:** Number and percentage of counties in the YRB's low-carbon economic development subregion (%).

Low carbon economic development sub-region	2020		2030		2060	
	Number	Proportion	Number	Proportion	Number	Proportion
CC-KOEZ	272	37.01	256	34.83	255	34.69
CC-APZ	228	30.75	212	28.84	211	28.71
CC-KEZ	144	19.59	95	12.93	97	13.20
CO-KOEZ	18	2.45	34	4.63	35	4.76
CO-APZ	6	1.09	22	2.99	23	3.13
CO-KEZ	67	9.11	116	15.78	114	15.51
ALL	735					

#### Carbon control zones

**Figure 8:**
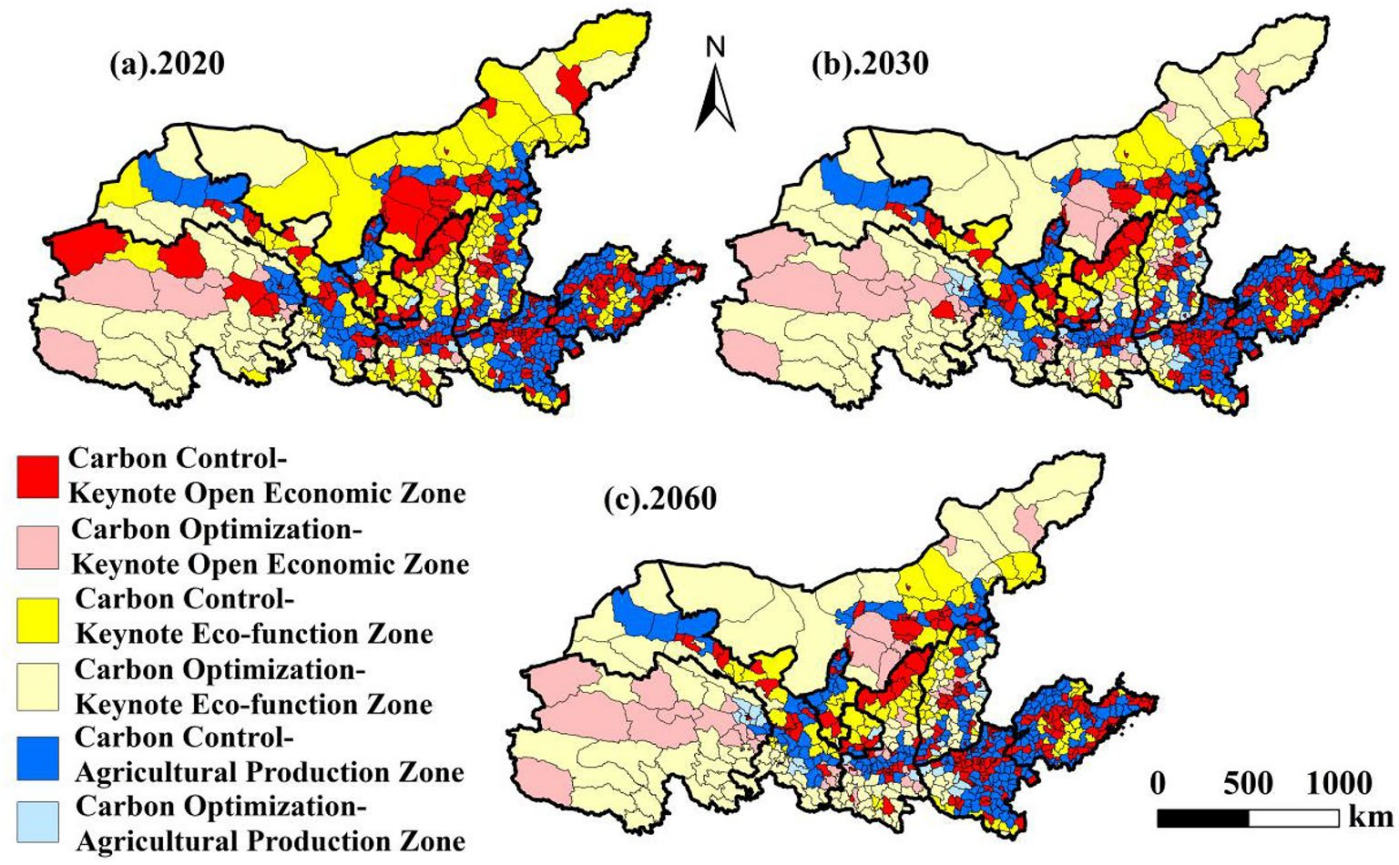
Sub-region of low-carbon economic development in the YRB.

Carbon control zones are much more numerous than carbon optimization zones, and contain key development zones, restricted main production zones of agricultural products and key ecological function zones in the region (Table 3). In 2020, the Carbon Control-Key Development Zone consists of 272 counties, including Baiyin District, Yindu District, Yongcheng City, Xiaodian District and Beilin District, accounting for 37.01% of the total number of counties in the YRB (Fig. 8a). Spatially, it is mainly distributed in the central Shandong Province, the region centered on Zhengzhou City in central Henan Province, the region centered on Taiyuan in Shanxi Province, the region of Hubao-Eyu urban agglomeration, the region of Hexi Corridor in Gansu Province, and the region centered on Xining City in Qinghai Province. Among them, there are 231 municipal districts and county-level cities, accounting for 84.93% of the number of CC-KDZ counties. The total carbon emissions in the region are 2.64 × 10^8^ t, accounting for 42.31% of the total carbon emissions in the CC-KDZ, and the average value of county carbon emissions reaches 96.89 × 10^4^ t. The number of counties in the CC-KDZ decreases in 2030 (Fig. 8b) and 2060 (Fig. 8c), and the total amount of carbon emissions is reduced by 43.56% and 40.15% relative to 2020, respectively. The average carbon emissions of counties within the region in 2030 and 2060 are 58.24 × 10^4^ t and 62.09 × 10^4^ t, which are 39.89% and 35.92% lower than in 2020, respectively.

The 2020 Carbon Control-Agricultural Products Main Production Zone (CC-APZ) consists of 226 counties, including Jingtai County, Maiyang County, Shangcai County, Dingtao County and Quwo County. Spatially the CC-APZ is opposite to the spatial distribution of the CC-KDZ, with the CC-APZ west of Shanxi almost coinciding with the main stream of the middle and upper reaches of the Yellow River, and distributed on both sides of the Fen River Basin within Shanxi Province, in the central-eastern plains of Henan Province, and in the hilly areas at the edges of Shandong Province. There are only 41 municipal districts in the CC-APZ, and the peripheral counties around the cities make up 81.86% of the total number of counties in the CC-APZ. The total carbon emissions in the region are 2.36 × 10^8^ t, accounting for 37.82% of the total carbon emissions in the Carbon Control Area (CC-APZ), and the average value of county carbon emissions reaches 110.81 × 10^4^ t. The number of counties in the CC-APZ decreases in 2030 and 2060, and the total amount of carbon emissions decreases by 41.53% and 39.83% relative to 2020, respectively. In 2030 and 2060, the counties in the region average carbon emissions are 65.29 × 10^4^ t and 67.43 × 10^4^ t, which are 41.08% and 39.15% lower than in 2020, respectively.

The 2020 Carbon Control-Key Ecological Functional Zones (CC-APZ) consists of 144 counties, including Huining County, Ulat Houqi, Jingyuan County, Bali County and Lingshi County. Spatially, the Key Ecological Functional Zones are distributed in the periphery of the main agricultural production areas, closer to the edge areas of the city than the CC-APZs, and discretely distributed in the Hexi Corridor area of Gansu Province, the grassland zone of northern Inner Mongolia, the provincial border counties of Shanxi, Shaanxi, Ningxia and Henan, and the hilly areas of the central part of Shandong Province. There are only 20 city districts and county-level cities, and the urban fringe counties account for 86.11% of the region. The total carbon emissions in the region are 1.04 × 10^8^ t, accounting for 19.87% of the total carbon emissions in the Carbon Control Area (CC-KEZ), and the average value of county carbon emissions reaches 72.09 × 10^4^ t. The number of CC-KEZ counties decreases in 2030 and 2060, and the total amount of carbon emissions decreases by 57.38% and 56.61% relative to 2020.The average values of county carbon emissions in 2030 and 2060 in the region are 46.67 × 10^4^t and 46.53 × 10^4^ t, which are 35% lower than 2020. Emissions average 46.67 × 10^4^ t and 46.53 × 10^4^ t, which are 35.26% and 35.77% lower than in 2020, respectively.

In conclusion, CC-KDZ, CC-APZ and CC-KEZ are gradually spreading from the city center to the periphery of the city, which is in line with the theory of "core-periphery" of urban spatial layout^38^. Carbon emissions per unit county CC-APZ > CC-KDZ > CC-KEZ. The rate of reduction of total carbon emissions per unit county in CC-APZ and CC-KDZ is basically the same as the total carbon emissions in the region. Under the future "dual carbon" goal, continue to control the total amount of carbon emissions to meet the needs of the region's total carbon emissions and the reduction of carbon emissions per unit of county. In the future, under the "dual carbon" target, it is necessary to focus on reducing the carbon emissions of individual counties in order to meet the demand for reducing the total amount of carbon emissions.

#### Carbon optimization zones

Carbon optimization zones also include key development zones in the region, main production zones of agricultural products with restrictions, and key ecological functional zones. 2020 carbon optimization—key development zones consists of 18 counties including Dengfeng City in Henan Province, Wendeng District in Shandong Province, the Xunhua Salar Autonomous County in Qinghai Province, Yaosu District, Xingping City, and Baota District in Shaanxi Province, and Jiaocheng County in Shanxi Province, which account for 2.45% of the total number of counties in the YRB (Fig. 8). Among them, city districts and county-level city districts accounted for 55.56% of the number of CO-KDZ counties. The net carbon absorption in the region is 37.02 × 10^4^ t, accounting for 10.54% of the total carbon emissions in the CO-KDZ, and the average value of carbon absorption in the counties is only 2.06 × 10^4^ t. The number of CO-KDZ counties increases to 34 and 35 in 2030 and 2060, and the total amount of carbon absorption increases by 1551.19 × 10^4^ t and 1532.28 × 10^4^ t relative to 2020 respectively. The average carbon sequestration of counties in the region in 2030 and 2060 is 46.71 × 10^4^ t and 52.67 × 10^4^ t, which is a significant increase from 2020.

The number of counties in the main carbon optimization-agricultural production zone (CO-APZ) in 2020 is less distributed, including only six urban fringe counties, including Heshui County, Ruyang County, Xiangcheng County, Zhongning County, Xia County, and Luonan County, accounting for 1.09% of the total number of counties in the YRB. The net carbon uptake in the region is 18.55 × 10^4^ t, and the average value of county carbon uptake is only 3.09 × 10^4^ t. The number of CO-APZ counties increases to 22 and 23 in 2030 and 2060, respectively, and the total amount of carbon uptake increases by 448.18 × 10^4^ t and 423.05 × 10^4^ t relative to 2020, respectively. The average value of county carbon emissions averaged 20.51 × 10^4^ t and 18.53 × 10^4^ t, a larger increase from 2020.

In 2020, Carbon Optimization—Key Ecological Functional Zones consists of 67 counties, including Diabe County, Abaga Banner, Heshun County and Liangdang County, accounting for 9.11% of the total number of counties in the YRB. Among them, there are only 2 municipal districts and county-level cities, namely Cooperation City and Yushu City. The net carbon absorption in the region is 328.93 × 10^4^ t, accounting for 85.55% of the total carbon emissions in the carbon optimization zone, and the average value of carbon absorption in counties is only 4.91 × 10^4^ t. The number of CO-KEZ counties increases to 116 and 114 in 2030 and 2060, respectively, which exceed the number of counties in the CC-KEZ. The total amount of carbon sequestration increases by 8330.02 × 10^4^ t and 8530.32 × 10^4^ t relative to 2020. The average values of carbon emissions from counties in the region in 2030 and 2060 are 74.65 × 10^4^ t and 77.71 × 10^4^ t, which is a significant increase from 2020.

In summary, CO-KDZ, CO-APZ and CO-KEZ have a more discrete spatial distribution due to their small overall numbers, and there is no obvious distribution pattern in space. Carbon emissions per unit county CO-KEZ > CO-APZ > CO-KDZ. the average value of carbon emissions from counties in CO-KEZ in 2030 and 2060 is much higher than that of other low-carbon economic development subregions. This suggests that the future low-carbon development of the YRB should give full play to the role of CO-KEZ, gradually increase the number of counties in CO-KDZ and CO-APZ, increase the total carbon absorption, and gradually reach the goal of carbon neutrality.

## Discussion

### Impact of land-use change on carbon balance

In this study, we estimated the land use carbon balance in 735 counties in the Yellow River Basin and found that the total carbon emissions decreased from 1980 to 2000, with a continuous increasing trend after 2000. This is consistent with the findings of Ren et al.^27^. The important reason for this is the rapid growth of carbon emissions since 2000, when China's industrialization spurt and the massive trade in fossil energy and resource fuels led to a substantial increase in domestic carbon emissions compared to the past. In the past 40 years, the area of construction land in the Yellow River Basin has increased by a net 4.98 × 10^4^ hm^2^ , the area of unutilized land has decreased by a net 4.78 × 10^4^ hm^2^ , and the area of all types of land transferred to construction land is as high as 12.42 × 10^4^ hm^2^ , which is much higher than that of the conversion between other land use types, and the conclusions of the status of the land use conversion are highly consistent with those of Meng et al.^50^. With the rapid development of urbanization, the urban population and the urban construction land area have increased significantly, leading to the continuous expansion of urban space, which in turn leads to an increase in carbon emissions of 3.21 × 10^8^ t. The increase in construction land, the increase in the intensity of human activities, the increase in the urban impermeable surfaces, and the resulting increase in the intensity of industrial production, and the accumulation and consumption of industrial energy fuels have brought about enormous environmental pressure, which are the main sources of carbon. Yang et al. showed that coal, oil, and natural gas account for 44%, 34%, and 21% of total carbon emissions, respectively. The sectors such as electricity, transportation and industry accounted for 38%, 24% and 23% of the total carbon emissions respectively, which in turn led to an increase in land use carbon emissions^21^.

Spatially, the carbon emissions from land use in the upper reaches of the Yellow River Basin are small, and the carbon emissions in the lower reaches are large, and the carbon emissions show an obvious characteristic of gradual decrease from east to west. The upstream area is rich in forest land, grassland and wetland resources, with high vegetation coverage, and these land uses are important ways of carbon sequestration^27^. The Chinese government's ecological projects such as “returning farmland to forests” and “Three-North Protective Forest Development Program” have greatly increased the forest cover through afforestation and mountain closure. The vegetation cover of the Loess Plateau increased from 31.6% in 2000 to 59.6% in 2015, mainly due to the reforestation of croplands with slopes of more than 15°^24^. Rapid industrialization in downstream areas has been accompanied by massive encroachment on grasslands, forests and wetlands, and industrial fossil fuels^42^. The increase in cropland and construction land due to the increase in urban land use and population are important sources of land use carbon emissions. Carbon sources have increased and carbon sinks have decreased, which in turn has led to much larger carbon emissions in the downstream than in the upstream areas.

Compared with 2020, the trend of land use carbon emissions in 2030 and 2060 is a gradual decrease in carbon emissions, a gradual increase in carbon absorption, and a gradual achievement of carbon neutrality^49^. Firstly, from the perspective of the development law of land use itself, the trend of land use transfer in the future is a decrease in arable land and an increase in the area of land for construction; secondly, the 2021 Outline of the Yellow River Basin Ecological Protection and High-Quality Development Plan emphasizes the strengthening of the upstream water source containment capacity construction, the middle reaches of soil and water conservation, and the downstream wetland protection and ecological management, and Qinghai should achieve carbon uptake first, whereas Shandong starts from the energy transformation and industrial upgrading Starting from, provinces with different levels of development have different future low-carbon development policies^51^. Finally, different economic development patterns in the upper, middle and lower reaches of the Yellow River Basin lead to different spatial layouts of cities, and human activities lead to changes in land use, which in turn affects changes in total carbon sources and sinks. Our findings remain in general agreement with those of Fan and Zhang et al.^6,22^.

### Optimization of carbon emission reduction potential and patterns in the YRB

In this study, we constructed a land-use carbon neutral budget model to distinguish the carbon control zone and carbon optimization zone in the Yellow River Basin. The area of the carbon emission sub-zone is much larger than the carbon absorption sub-zone in 1980–2020, and the net carbon emission is much larger than the net carbon absorption. This is consistent with the results of Wang et al.^52^. The high value area of the carbon control zone shows a decreasing trend from southeast to northwest, and the high value is along the main stream of the Yellow River. This is mainly due to the fact that the upstream river valleys and plain areas along the river are suitable for urban construction and development, resulting in the expansion of the construction land area, which leads to an increase in carbon sources, while the mountainous plateau areas have high vegetation coverage and higher carbon sinks, making a surplus of carbon emissions and a high potential for carbon emission reduction. Bounded by the Qilian Mountains, the eastern part of the region is dominated by plains and hills, which are suitable for urban development, leading to the concentration of high value areas for carbon control in Shandong and Henan provinces. Shanxi, Shaanxi and southern Ningxia are carbon control low-value areas with more serious carbon emission deficits and small carbon reduction potential. Therefore, the carbon emission reduction potential of the Yellow River Basin is spatially characterized by “low in the southeast and high in the northwest”, with a large spatial difference in carbon emission reduction potential^53^.

In terms of total provincial carbon emissions, in 2020, the total net carbon emissions of Shandong Province (2028.63 × 10^4^ t) and Henan Province (1722.84 × 10^4^ t) will be 3751.47 × 10^4^ t, and the net carbon emissions of Ningxia and Gansu (738.16 × 10^4^ t). Therefore, Shandong and Henan provinces should take the lead in optimizing the carbon emission reduction pattern. Shandong Province's energy structure is dominated by high-carbon fossil energy, of which fossil energy accounts for about 88%, ranking first in the country. Coal consumption accounts for more than 76% of non-renewable energy consumption. All-out efforts are being made to promote the optimization and adjustment of the three major structures of energy, industry and transportation, and the biased industrial structure requires accelerating the optimization and upgrading of traditional industries and promoting the construction of a green financial system in the forefront^54^. In Henan Province, by 2025, the province's energy consumption per unit of GDP will be reduced by more than 14.5% compared with 2020, the total energy consumption will grow reasonably, green planning, green construction and green operation and management of cities and towns will be comprehensively promoted, sponge city construction will be systematically promoted in the whole region, and the construction of resilient cities and “no-waste cities” will be accelerated^55^. Support wind, solar, biomass and other renewable energy substitution, and orderly promote clean heating in rural areas. In the future, under the dual-carbon goal, we should build carbon emission reduction pioneering zones mainly in Shandong and Henan, and carbon optimization demonstration zones mainly in Qinghai, Gansu, and Ningxia, so as to improve carbon absorption and promote carbon neutrality in terms of low-carbon economic development.

### Low carbon economic development models in different regions of the YRB

This study superimposes the main functional area planning on the basis of carbon neutral zoning, and divides the low carbon economic development zoning of the Yellow River Basin into 6 levels.CC-KDZ is concentrated in the municipal districts and county-level cities, and the carbon emissions in this region account for 42.31% of the total carbon control area, with the smallest carbon emission reduction potential, and the future in the Carbon Control-Key Development Zones, which should lead the direction of carbon emission reduction. Due to the high level of economic development in CC-KDZ, high population density, urban construction land area accounts for a relatively large area, improve the implementation of dual control of energy consumption intensity and total volume is an important mode of low-carbon economic development in the future CC-KDZ. Establishment of green transformation and upgrading of key industries, energy saving and emission reduction in transportation and logistics, and green energy saving projects in urban and rural areas. Improve the key regional pollutant emission reduction, volatile organic compounds comprehensive management system, and improve the level of environmental infrastructure. Give full play to the key economic, cultural and social development functions of CC-KDZ in the ecological protection and high-quality development of the Yellow River Basin, and ensure that the province realizes carbon peaking by 2030.

CC-APZ is distributed in the periphery of CC-KDZ, and as the main agricultural production area of the Yellow River Basin, cropland is one of the largest carbon sources, and there are challenges to the synergistic development of carbon sequestration and emission reduction in cropland system and food security. First, efficient allocation of agricultural input factors. According to the regional resource endowment and agricultural development situation, guide the efficient allocation of labor, technology, capital and other production factors among food functional areas. Second, improve the incentive mechanism of ecological compensation for arable land utilization. Link the environment-friendly cropland utilization mode with the ecological compensation policy, moderately increase the amount of subsidies for farmers to use organic fertilizers and low-carbon pesticides, and stimulate the enthusiasm of farmers to use cropland in a low-carbon manner. Finally, increase investment in agricultural science and technology research and development. Research and development of cropland system ecological management technology, repair cropland ecosystems, improve the quality of cropland ecosystems, and innovate cropland carbon sequestration and emission reduction technology^56^. Accelerate the construction of agricultural carbon emission accounting method system. As soon as possible to form a set of accounting method system recognized by the management department, business subjects and carbon trading subjects, to lay the foundation for agriculture to enter the carbon market.

CC-KEZ is distributed in the periphery of the main agricultural products producing areas and the inter-provincial border area. As it is in the carbon control zone, carbon emissions are much larger than the total carbon absorption, the key ecological function zones prioritize the protection of ecology, and emission reduction is an important mode for the future development of low-carbon economy in the region. First, establish a sound categorized compensation system. Strengthen the conservation of aquatic biological resources and ensure that the 10-year fishing ban in key waters of the Yangtze River Basin is put into effect. For the river headwaters, important water sources, key soil erosion prevention and control areas, flood storage areas, damaged rivers and lakes and other key areas to carry out water flow ecological protection compensation^57^. Second, highlight the focus of vertical compensation. On the Qinghai-Tibet Plateau, the South-to-North Water Diversion water source areas and other ecological functions of outstanding importance, in the key ecological functional area transfer payment measurement by increasing the transfer coefficient, plus ecological and environmental protection expenditures and other ways to increase support, and promote its ability to guarantee basic public services to the forefront of the same level of financial resources in the region. Finally, explore diversified compensation methods. Support ecological functionally important areas to carry out ecological and environmental protection education and training, guide the development of characteristic advantageous industries, expand the production of green products, and accelerate the development of ecological agriculture and recycling agriculture^58^.

CO-KDZ focuses on economic development under the premise of greater carbon absorption. Based on the carrying capacity of the resources and environment, give full play to the comparative advantages of CO-KDZ counties, promote the reasonable flow and efficient concentration of various types of factors, and promote the formation of a new pattern of territorial spatial development and protection with obvious main functions, complementary advantages, and high-quality development. This is an important model for future low-carbon economic development in CO-KDZ. It will provide CC-KDZ counties with experience and technology in controlling carbon emissions, and drive the synergistic development of CC-KDZ while developing its own low-carbon economy.

As the CO-APZ has the smallest number and belongs to urban fringe counties, it can accommodate the migration of agricultural products from the carbon control zone within the ecological carrying capacity threshold, extend the agricultural industry chain, optimize and strengthen the agricultural products processing industry and the agricultural production service industry, and absorb more of the county's agricultural transfer population, so as to provide support for the effective service of the “Three Rural Areas” and safeguard food security. Provide support to effectively serve the “three rural areas” and guarantee food security.

The number of counties in the CO-KEZ exceeds that of the CC-KEZ in the forecast results for 2030 and 2060. In the future, the development of counties in key ecological functional zones should be carried out in an orderly manner, and CC-KEZ counties should be gradually converted into CO-KEZ counties. It will promote counties located in key ecological functional zones to gradually and orderly undertake the transfer of overloaded population from ecological areas, improve the financial transfer system, enhance the supply capacity of public services, and develop suitable industries and clean energy, so as to provide support for the protection and restoration of ecological environments and the building of ecological security barriers.

### Limitations and prospects of the study

Our study has some limitations. First, there is a certain error in our prediction of future land use. This error comes from the research data and methodology, the simulation of PLUS model is not yet able to fully restore the real land use changes, the future population, climate, urban economic and social development changes can not be predicted in fine detail, the uncertain objective factors lead to the inevitable problems in the modeling research method. Secondly, the change of people's values is not predicted in our research, the improvement of human awareness of ecological protection and the improvement of the adaptive capacity of the economic environment will have an important impact on the development of our low carbon economy in the future. Overall, despite our shortcomings in the above areas, we compared and frontier scholars' conclusions and tested the results of our prediction model to achieve a relatively good Kappa coefficient test value, thus the results of this study are generally reliable. In the future research, we need to further improve the PLUS model to integrate the land use scenarios in different contexts into the process we need to study, together with the superposition of many statistical indicator elements, to add more accurate and scientific theoretical research support for our research results. At the same time, we are currently planning to optimize the division of the main functional zoning, in the future, we also need to put into practice, the study of spatial planning of the land combined with the existing empirical basis, to come up with more practical conclusions.

## Conclusion

This study evaluates the spatial and temporal evolution of carbon revenues and expenditures in 735 counties in the YRB from 1980 to 2020 by means of the carbon revenue and expenditure model, and utilizes the PLUS prediction model for the spatial pattern of land-use carbon revenues and expenditures in 2030 and 2060, and at the same time superimposes the main functional area planning to divide the low-carbon economic development sub-area in the YRB, and obtains the following main conclusions:

1. The spatial and temporal changes in land use in the YRB from 1980 to 2020 are large. The area of cultivated and unutilized land decreases, and the area of other land types increases. The net transfer area of construction land is the largest, which is twice as large as the total net increase of other land use types. In 2030 and 2060, relative to 2020, the land use types with the largest number of net transfers of land use in the natural scenario are both construction land, followed by unutilized land.
2. The spatial and temporal distribution pattern of carbon neutrality from 1980 to 2020 is mainly influenced by the carbon emissions of each county, and the net carbon emissions of each county are much larger than the net carbon absorption. The spatial and temporal distribution characteristics of carbon neutral and carbon emissions are basically consistent, showing a decreasing trend from southeast to northwest. The spatial and temporal distribution of carbon absorption is the opposite, showing a trend of high in the west and low in the east, and the spatial distribution of carbon emission, carbon absorption and carbon neutrality in the years 2030 and 2060 is basically consistent with the spatial distribution characteristics in 1990.
3. The low-carbon economic development zones in the YRB show obvious spatial differentiation characteristics. The number of counties in the Carbon Control Zone is much larger than that in the Carbon Optimization Zone, among which the number of counties in the CC-KOEZ is the largest, followed by the CC-APZ. The future development of low-carbon economy in the YRB has different development modes for different subzones (Supplementary Information).

### Supplementary Information

Supplementary Information 1.

Supplementary Information 2.

Supplementary Information 3.

Supplementary Information 4.

Supplementary Information 5.

Supplementary Information 6.

## Data Availability

The datasets used and/or analysed during the current study available from the corresponding author on reasonable request.

## References

[1] Allen MR, Frame DJ, Huntingford C (2009). Warming caused by cumulative carbon emissions towards the trillionth tonne. Nature.

[2] Melillo JM, Steudler PA, Aber JD (2002). Soil warming and carbon-cycle feedbacks to the climate system. Science.

[3] Song J, Du JW, Wang F (2023). Carbon emission and industrial structure adjustment in the Yellow River Basin of China: Based on the LMDI decomposition model. Nat. Environ. Pollut. Technol..

[4] Yongxian Su, Xiuzhi C, Yuyao Ye (2013). The characteristics and mechanisms of carbon emissions from energy consumption in China using DMSP/OLS night light imageries. Acta Geogr. Sin..

[5] Birner B, Rödenbeck C, Dohner JL (2023). Surprising stability of recent global carbon cycling enables improved fossil fuel emission verification. Nat. Clim. Chang..

[6] Chung MG, Frank KA, Pokhrel Y (2021). Natural infrastructure in sustaining global urban freshwater ecosystem services. Nat. Sustain..

[7] Houghton RA, Hackler JL, Lawrence KTT (1999). The U.S. carbon budget: Contributions from land-use change. Science.

[8] Strengers BJ, Van Minnen JG, Eickhout B (2008). The role of carbon plantations in mitigating climate change: Potentials and costs. Clim. Change.

[9] Houghton R (2012). Carbon emissions and the drivers of deforestation and forest degradation in the tropics. Curr. Opin. Environ. Sustain..

[10] Huang Y, Shen L, Liu H (2019). Grey relational analysis, principal component analysis and forecasting of carbon emissions based on long short-term memory in China. J. Clean. Prod..

[11] Le Noë J, Erb K-H, Matej S (2021). Altered growth conditions more than reforestation counteracted forest biomass carbon emissions 1990–2020. Nat. Commun..

[12] Zhonghua C, Lan W, Yi Z (2022). Does smart city policy promote urban green and low-carbon development?. J. Clean. Prod..

[13] Tang X, Zhang W, Lin W (2020). Low-carbon sustainable development of China's manufacturing industries based on development model change. Sci. Total Environ..

[14] Wall MA, Laubach J, Campbell ID (2024). Effects of dairy farming management practices on carbon balances in New Zealand’s grazed grasslands: Synthesis from 68 site-years. Agric. Ecosyst. Environ..

[15] Yang Y, Zhu Y, Zhao Y (2024). Improving farmers’ livelihoods through the eco-compensation of forest carbon sinks. Renew. Sustain. Energy Rev..

[16] Dong H, Hu Y, Qian L (2024). Preliminary manifestation of the Yangtze River Protection Strategy in improving the carbon sink function of estuary wetlands. iScience.

[17] Jing PR, Sheng JB, Hu TS, Mahmoud A, Guo LD, Liu Y, Wu YT (2022). Spatiotemporal evolution of sustainable utilization of water resources in the Yangtze River economic belt based on an integrated water ecological footprint model. J. Clean. Prod..

[18] Praveen S, Niraj M, Nripendra S (2024). Beyond carbon footprints: the ‘Greta Thunberg effect’ and tourist hotel preferences. J. Travel Tour. Market..

[19] Fang M, Tan SK, Wirjanto ST (2024). Valuation of carbon emission allowance options under an open trading phase. Energy Econ..

[20] Li Y, Zhang Z, Zhao Z (2023). Zoning prediction and mapping of three-dimensional forest soil organic carbon: A case study of subtropical forests in southern China. Forests.

[21] Runjia Y, Hong C, Sha C (2022). Spatiotemporal evolution and prediction of land use/land cover changes and ecosystem service variation in the Yellow River Basin, China. Ecol. Indic..

[22] Gang X, Jingling S, Chang X (2022). Spatial mismatches between nighttime light intensity and building morphology in Shanghai, China. Sustain. Cities Soc..

[23] Tao W, Xiaoyi W, Dan L (2023). The current and future of terrestrial carbon balance over the Tibetan Plateau. Sci. China Earth Sci..

[24] Wang J, Liu Y, Wang S (2024). Enhanced ecosystem carbon sink in shrub-grassland ecotone under grazing exclusion on Tibetan plateau. Ecol. Indic..

[25] Siyou X, Yu Y (2022). Examining spatio-temporal variations in carbon budget and carbon compensation zoning in Beijing-Tianjin-Hebei urban agglomeration based on major functional zones. J. Geogr. Sci..

[26] Li P, Chen J, Li Y (2023). Using the InVEST-PLUS model to predict and analyze the pattern of ecosystem carbon storage in Liaoning Province, China. Remote Sens..

[27] Chen J, Wang K, Li M, Wang X, Zhang X, Niu L, Zhang Y (2023). Prediction and evolution of carbon storage of terrestrial ecosystems in the Qinling mountains North Slope Region, China. Land..

[28] Meng W, Changzheng Z, Ying C (2022). The influencing factors of carbon emissions in the railway transportation industry based on extended LMDI decomposition method: evidence from the BRIC countries. Environ. Sci. Pollut. Res. Int..

[29] Yonghua L, Hezhou J, Bo Z (2023). Comparative evaluation of multi-scale spatiotemporal variability and drivers of carbon storage: An empirical study from 369 cities, China. Ecol. Indic..

[30] Marco P, Beatriz M (2022). Soil management and compost amendment are the main drivers of carbon sequestration in rainfed olive trees agroecosystems: An evaluation of chemical and biological markers. Catena.

[31] Tianqi R, Pengyan Z, Huiru Z (2022). Spatial correlation evolution and prediction scenario of land use carbon emissions in China. Ecol. Inform..

[32] Jiqiang L, Xianghang F, Chen L (2023). Quantitative assessment of spatiotemporal dynamics in vegetation NPP, NEP and carbon sink capacity in the Weihe River Basin from 2001 to 2020. J. Clean. Prod..

[33] Wen L, Xin Z, Qi F (2023). Analyzing the impacts of topographic factors and land cover characteristics on waterlogging events in urban functional zones. Sci. Total Environ..

[34] Jiale H, Afeng Z, Yanhong K (2022). Biochar promotes soil organic carbon sequestration and reduces net global warming potential in apple orchard: A two-year study in the Loess Plateau of China. Sci. Total Environ..

[35] Qiufeng Z, Junfeng L, Yue L (2023). Coupling analysis and driving factors between carbon emission intensity and high-quality economic development: Evidence from the Yellow River Basin, China. J. Clean. Prod..

[36] Wang X, Zhao X, Zhang S (2023). Decoupling effect and driving factors of land-use carbon emissions in the Yellow River Basin using remote sensing data. Remote Sens..

[37] Chenglong X, Qibin Z, Qiang Y (2023). Effects of land use/cover change on carbon storage between 2000 and 2040 in the Yellow River Basin, China. Ecol. Indic..

[38] Zhang Z, Liu L, Zhang J (2023). Study on urban spatial expansion and its scale benefit in the Yellow River Basin. Sustainability.

[39] Zhongwu Z, Jinyuan Z, Liping L (2023). Spatial-temporal heterogeneity of urbanization and ecosystem services in the Yellow River Basin. Sustainability.

[40] Fubo Z, Xi W, Shuai M (2023). Widespread increasing ecosystem water limitation during the past three decades in the Yellow River Basin, China. J. Geophys. Res. Biogeosci..

[41] Kai L, Liping F, Fangbai L (2021). Sustainability assessment and carbon budget of chemical stabilization based multi-objective remediation of Cd contaminated paddy field. Sci. Total Environ..

[42] Lin Z, Chuanhao Y, Yuchen Z (2023). Spatial correlations of land use carbon emissions in Shandong peninsula urban agglomeration: a perspective from city level using remote sensing data. Remote Sens..

[43] Huijun W, Kanglong D, Zhanfeng D (2022). Comprehensive assessment of land use carbon emissions of a coal resource-based city, China. J. Clean. Product..

[44] Qianmin J, Jia W, Shahzad A (2020). Nutrient management and cultivation techniques affect maize production through regulating greenhouse gas intensity and carbon budget under semi-arid climate. J. Clean. Prod..

[45] Lina G, Fei T, Runrui L (2022). Multi-scenario simulation and ecological risk analysis of land use based on the PLUS model: A case study of Nanjing. Sustain. Cities Soc..

[46] Haight JD, Hall SJ, Fidino M (2023). Urbanization, climate and species traits shape mammal communities from local to continental scales. Nat. Ecol. Evol..

[47] Shuai S, Yong Y (2023). Identification of ecological improvement zones in different ecological functional zones in northwest Hubei, China. Ecol. Indic..

[48] Tingting P, Fenzhen S, Fengqin Y (2023). Optimization of multi-objective multi-functional landuse zoning using a vector-based genetic algorithm. Cities.

[49] Shisheng L, Hui G, Haibo Z (2023). The fate of antibiotic resistance genes in the coastal lagoon with multiple functional zones. J. Environ. Sci..

[50] Junxiong M, Piling S, Dandan S (2023). The dynamic patterns and driving factors of land use conflict in the Yellow River basin of China. Environ. Sci. Pollut. Res. Int..

[51] Yifang S, Ninglian W (2022). Evolution and obstacle factors of high-quality industrial development in the π-shaped Curve Area of the Yellow River basin in China. J. Geogr. Sci..

[52] Shiyi W, Yan L, Feng L (2023). Spatialization and driving factors of carbon budget at county level in the Yangtze River Delta of China. Environ. Sci. Pollut. Res. Int..

[53] Fred WM (2022). Constraining the carbon budget of peat ecosystems: Application of stoichiometry and enthalpy balances. J. Geophys. Res. Biogeosci..

[54] Zhang S, Wang M, Zhu H (2024). Impact factors and peaking simulation of carbon emissions in the building sector in Shandong Province. J. Build. Eng..

[55] Liu J, Li B, Ma M (2024). Spatiotemporal variation and causes of typical extreme precipitation events in Shandong Province over the last 50 years. Remote Sens..

[56] Sohini G, Rituparna B, Sunanda B (2022). Carbon sequestration and greenhouse gas emissions for different rice cultivation practices. Sustain. Prod. Consum..

[57] Xiaozhen W, Jianzhao W, Yulin L (2022). Driving factors of ecosystem services and their spatiotemporal change assessment based on land use types in the Loess Plateau. J. Environ. Manag..

[58] Yuan Z, Zhen Y, Juan Z (2022). Spatiotemporal evolution characteristics and dynamic efficiency decomposition of carbon emission efficiency in the Yellow River Basin. PloS one.

